# A method to estimate cell cycle time and growth fraction using bromodeoxyuridine-flow cytometry data from a single sample

**DOI:** 10.1186/1471-2407-5-122

**Published:** 2005-09-22

**Authors:** Rimantas Eidukevicius, Dainius Characiejus, Ramunas Janavicius, Nijole Kazlauskaite, Vita Pasukoniene, Mykolas Mauricas, Willem Den Otter

**Affiliations:** 1 Faculty of Mathematics and Informatics, Vilnius University, Naugarduko 24, 03225 Vilnius, Lithuania; 2 Institute of Oncology, Vilnius University, Santariškių 1, 08660 Vilnius, Lithuania; 3 Institute of Immunology, Vilnius University, Moletų pl. 29, 08409 Vilnius, Lithuania; 4 Department of Pathobiology, Utrecht University, P.O. Box 80158, 3508 TD Utrecht, The Netherlands

## Abstract

**Background:**

Presently available flow cytometric methods of bromodeoxyuridine (BrdUrd) labelling do not provide information on the cell cycle time (T_C_) and the growth fraction (GF). In this paper, we describe a novel and simple method to estimate T_C _and GF from flow cytometric analysis of a single tumour sample after BrdUrd labelling.

**Methods:**

The proposed method is based on two assumptions: (1) the number of labelled cells traversing the cell cycle per unit time is constant and (2) the total number of labelled cells is constant throughout the cycle, provided that cells produced after division are excluded. The total numbers of labelled divided G_1 _cells, labelled divided S cells, labelled undivided S cells, and labelled undivided G_2 _cells were obtained for DNA histograms of BrdUrd-positive cells in a collected sample. These cell numbers were used to write equations to determine the durations of cell cycle phases, T_C _and GF. To illustrate the application of the proposed formulae, cell cycle kinetic parameters were analysed in solid SL2 tumours growing in DBA/2 mice and in human T-leukaemia Jurkat cells in culture.

**Results:**

The suitability of the proposed method for estimating durations of the cell cycle phases, T_C _and GF was demonstrated. T_C _in SL2 tumours was found to be relatively constant at 4 and 10 days after tumour implantation (20.3 ± 1.1 h and 21.6 ± 0.9 h, respectively). GF in tumours at day 10 was lower than GF at day 4 (54.2 ± 7.7% vs. 79.2 ± 5.9%, p = 0.0003). Approximate values of T_C _and GF of cultured Jurkat cells were 23.9 h and 79.3%, respectively.

**Conclusion:**

The proposed method is relatively simple and permits estimation of the cell cycle parameters, including T_C _and GF, from a single tumour sample after labelling with BrdUrd. We have shown that this method may be useful in preclinical studies, allowing estimation of changes in GF during growth of murine tumours. Experiments with human Jurkat cells suggest that the proposed method might also prove suitable for measurement of cell kinetics in human tumours. Development of suitable software enabling more objective interpretation of the DNA profile in this method would be desirable.

## Background

Uncontrolled proliferation is regarded as one of the most important traits of malignancy. Therefore, a considerable effort has been directed to the evaluation of the parameters of cell kinetics in tumours. A significant improvement in cell kinetic studies was achieved by the introduction of the technique of flow cytometric analysis of cells labelled with bromodeoxyuridine (BrdUrd) and the relative movement (RM) method [1]. The RM method allows estimation of the duration of S phase (T_S_) and the labelling index (LI) from a single sample. From these parameters, the potential doubling time (T_pot_) can be calculated. T_pot _is defined as the time to double the number of proliferating cells in the absence of cell loss [1]. This derived cell kinetic parameter has been postulated to be a predictor of a tumour's proliferative capability and has been widely studied in clinic in an attempt to find its prognostic significance and the potential value in predicting treatment outcome [2]. However, more recent evidence has not confirmed the existence of association between T_pot _and disease outcome measures, whereas information on clinical significance of LI and T_S _remains ambiguous [3-5]. Therefore, new methods of evaluating cell kinetics in tumours are required.

Cell production rate is determined by the cell cycle time (T_C_), i.e. the time interval between cell divisions, and the growth fraction (GF), i.e. the proportion of cells engaged in the cell cycle. Thus, T_C _and GF can be regarded as the most important parameters in evaluating cell production in tumours. However, neither the original RM method [1] nor its more recent modification [6] provide enough data to calculate the duration of all cell cycle phases, consequently the duration of the whole cycle, and GF of a tumour.

T_C _and GF can be estimated by calculating the percent BrdUrd labelling in tumour samples vs. time after injection of BrdUrd. However, the disadvantage of this method is that subsequent tumour samples have to be taken at intervals during several days after the administration of BrdUrd [7]. This method does not allow a measurement of cell kinetics in an individual tumour at a particular time point and is useless in a clinical setting.

Currently, proliferation-related antigen Ki-67 is widely used to measure GF in tumours and normal tissues. The Ki-67 antigen is considered to be present in the nuclei of cells in the G_1_, S and G_2 _phases of the cell cycle as well as in mitosis, but not in quiescent cells (cells in G_0_) [8]. Although the relationship between Ki-67 protein expression and cell proliferation is established, there is some evidence that expression of Ki-67 antigen may be minimal in late G_1 _and early S phase cells [9]. The accessibility of Ki-67 epitopes may vary during the cell cycle [10]. In addition, not all cells containing Ki-67 antigen may be actively proliferating [11]. Thus, detection of proliferative markers (such as Ki-67) may not correspond in every case to the theoretically defined term of GF. Obviously, determination of proliferative markers does not provide information about T_C_.

In this paper, we describe a novel and simple method to estimate T_C _and GF from flow cytometric analysis of a single tumour sample after BrdUrd labelling.

## Methods

### Mice and tumours

Female DBA/2 mice at the age of 8–12 weeks were obtained from the local breeding facility at the Institute of Immunology, Vilnius, Lithuania. SL2, a spontaneously arisen DBA/2 – derived lymphoma was maintained by weekly intraperitoneal passage in DBA/2 mice. Solid SL2 tumours for cell proliferation analysis were induced by subcutaneous injection of 10^7 ^SL2 cells on the chest of mice and analysed after 4 and 10 days.

Experimental research on animals has been conducted according to recommendations of the Lithuanian Ethics Committee for the Laboratory Animal Use.

### Cell culture

Human acute T-cell leukaemia Jurkat cells were cultured in RPMI 1640 medium supplemented with 10% foetal bovine serum. The cultures were incubated at 37°C in a humidified atmosphere with 5% CO_2_.

### BrdUrd labelling

The mice were injected i.p. with 1 ml of 2 mg/ml BrdUrd (5-bromo-2'-deoxyuridine, Sigma, St Louis, MO, USA) solution in physiological saline. The half-life of BrdUrd in rodent blood is only about 15 min. Therefore, a single i.p. injection acts as a pulse label [12]. At 10 h after BrdUrd injection the tumours were dissected and cut into slices of about 1 mm thickness. To avoid sampling errors, several slices were cut both from central and peripheral parts of a tumour. Tumour slices were fixed in 70% ethanol, and stored at +4°C for one or two days until staining.

For in vitro labelling of Jurkat cells, BrdUrd was dissolved in phosphate-buffered saline (PBS) and added to culture medium at a final concentration of 10 μM. After incubation for 30 min at 37°C, the cells were rinsed twice with RPMI 1640 medium and the cultures further incubated at 37°C. Samples for analysis were taken at 8, 10, 12 and 16 h after the BrdUrd pulse, the cells were washed with PBS and fixed in 70% ice-cold ethanol.

### Flow cytometric staining and measurement

Slices of solid SL2 tumours were cut in fragments of about 1 mm^3 ^and incubated in 0.4 mg/ml pepsin solution in 0.1 N HCl at 37°C with continuous agitation to produce a nuclear suspension. Isolation of nuclei was revealed by microscopic observation after 30 to 60 min of incubation. The nuclear suspension was filtered through a 48 μm nylon mesh. Nuclear suspensions of SL2 cells and suspensions of fixed Jurkat cells were washed and incubated in 2 N HCl for 30 min at room temperature for DNA denaturation. The acid solution was then neutralised with 0.1 M Na_2_B_4_O_7 _(pH 8.5) and the nuclear or cell suspensions were washed twice in PBS. Fifty μl of PBS containing 0.5% Tween 20 and 20 μl of FITC-conjugated mouse anti-BrdUrd monoclonal antibody (Becton Dickinson, Heidelberg, Germany) was added to the pellet containing 10^6 ^nuclei and incubated for 30 min at room temperature in the dark. After washing twice in PBS, 1 ml of a solution containing 5 mg/l propidium iodide (Sigma, St Louis, MO, USA) in PBS was added to the pellet and incubated for 30 min at 37°C in the dark.

The cellular DNA content and the amount of incorporated BrdUrd were simultaneously measured using a FACSort flow cytometer (Becton Dickinson, Heidelberg, Germany). Green fluorescent light emission (FITC = BrdUrd incorporation) was collected in the FL1 detector and red fluorescence (propidium iodide = DNA content) was collected in FL2 (for SL2 cells) or FL3 (for Jurkat cells) detector. Data from 15,000 nuclei per sample were acquired as dot plots of BrdUrd labelling vs. DNA content using LYSYS II software (Becton Dickinson, Heidelberg, Germany). Doublets were excluded by using the doublet discrimination mode. WinMDI version 2.8 software was used to analyse the acquired data.

### Mathematical formulae for estimation of duration of cell cycle phases and GF in BrdUrd-labelled tumours

**Figure 1:**
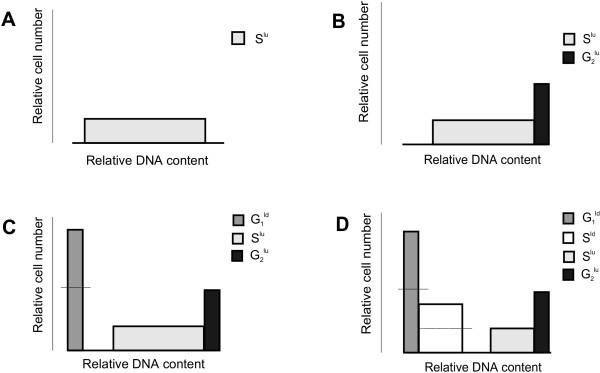
**Theoretical DNA distributions of BrdUrd-labelled cells *vs*. cell number at different post-labelling times**. A – immediately after BrdUrd pulse, i.e. at t = 0, B – at 0 < t <, C – at  < t < T_S _and  < t < + , and D – at  +  < t < T_S_. , T_S _and  are durations of G_1_, S and G_2 _phases of the cell cycle, respectively. Horizontal lines dividing G_1_^ld ^and S^ld ^bars indicate that half of the labelled divided cells are excluded from calculations.

We propose that the total number of cells S^l ^labelled during the BrdUrd pulse can be estimated after any time interval shorter than the duration of the cell cycle as



where S^lu ^– labelled undivided S cells, G_2_^lu ^– labelled undivided G_2 _cells, G_1_^ld ^– labelled divided G_1 _cells, and S^ld ^– labelled divided S cells (1/2 is introduced to exclude cells produced after division). Theoretical DNA distributions of BrdUrd-labelled cells at different post-labelling times are graphically represented in Figure 1. The equation (1) is based on the assumption that labelled cell mortality or exit from the cell cycle during the period of measurement is negligible. If this is the case, the total number of cells labelled during the BrdUrd pulse remains constant throughout the cycle, provided that cells produced after division are excluded. Mitotic cells are not included in the analysis, since they lack the nuclear membrane and the cell membrane is disrupted by the nuclear isolation medium used for preparation for flow cytometric analysis [13]. Taking into account that the mitotic time is relatively short and the number of cells in mitosis can be regarded as negligible, the omission of mitotic cells may not have a considerable impact on estimation of the total number of BrdUrd-labelled cells.

**Figure 2:**
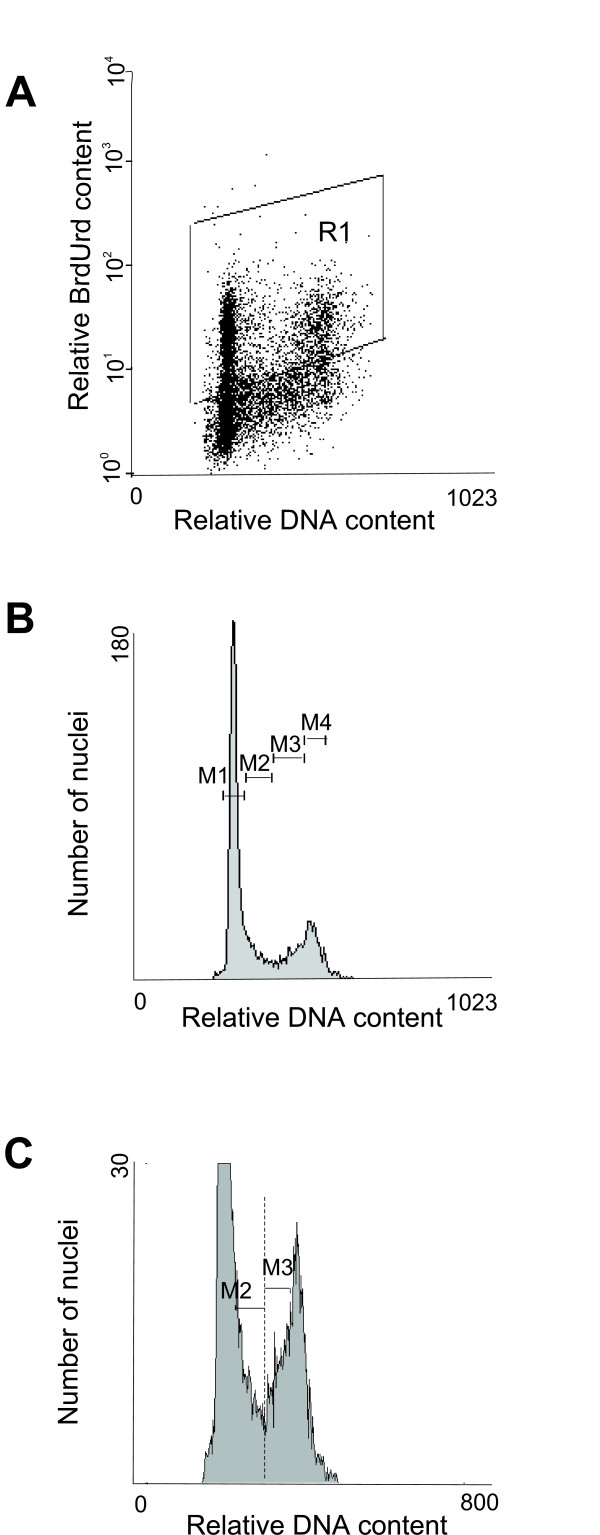
**Example of estimation of SL2 tumour cell numbers within different compartments of the cell cycle 10 h after *in vivo *labelling with BrdUrd**. A. Flow cytometric dual parameter dot plot of BrdUrd content (log FITC fluorescence) vs. DNA content (propidium iodide fluorescence) with a gate set for BrdUrd positive cells. B. DNA histogram analysis of BrdUrd positive cells; M1 indicates labelled divided G_1 _cells (G_1_^ld^); M2 indicates labelled divided S cells (S^ld^); M3 indicates labelled undivided S cells (S^lu^); M4 indicates labelled undivided G_2 _cells (G_2_^lu^). C. The same DNA histogram as in B, shown at higher magnification; S^ld ^compartment is separated from S^lu ^compartment by the channel corresponding to the lowest number of events (indicated by dotted vertical line). Total number of cells within each of these markers is obtained using statistics option of WinMDI 2.8 software.

Dot plots of BrdUrd labelling vs. DNA content were used to gate BrdUrd-positive cells (Figure 2A). The gate settings were adjusted on the control profiles, so as reproducibly to distinguish BrdUrd-positive fluorescence. G_1_^ld^, S^ld^, S^lu ^and G_2_^lu ^compartments in each DNA histogram of BrdUrd-positive cells were determined manually and numbers of cells in each of these compartments were obtained using statistics option of WinMDI 2.8 software (Figure 2B). These cell numbers were used in the equations of the proposed method. Instead of cell numbers, cell percentages over the total number of cells measured per sample can also be used.

The proposed method requires that considerable proportions of cells be found in S^lu ^and S^ld ^compartments. S^lu ^and S^ld ^compartments can be distinguished in the DNA histogram of BrdUrd-positive cells, provided that the time interval t after the BrdUrd pulse is shorter than the duration of the S phase, but sufficient for the labelled cells to re-enter a new S phase, i.e. if  +  < t < T_S _(,  and T_S _are durations of G_2_, G_1 _and S phases of the cell cycle, respectively). S^ld ^compartment was separated from S^lu ^compartment by the channel corresponding to the lowest number of events (Figure 2C).

The number of labelled cells traversing S phase per unit time can be estimated as



The number of labelled cells traversing G_2 _phase, mitosis, G_1 _phase and re-entering S phase per unit time can be estimated as



The labelled cells begin to enter G_1 _phase after time  and the number of cells traversing G_1 _phase and re-entering S phase per unit time can be estimated as



The labelled cells begin to re-enter S phase after time  +  and the number of cells re-entering S phase per unit time can be estimated as



Estimation of durations of cell cycle phases in the proposed method is based on the assumption that the number of labelled cells traversing the cell cycle per unit time is constant. One of the models illustrating this assumption is the rectangular age distribution of proliferating cells described by Steel [14]. In this case, ν_1 _= ν_2 _= ν_3 _= ν_4 _and



The equation (1) and sequentially the equalities of the equation (6) can be used to determine T_S_,  and :







GF can be estimated using the classical formula reported by Steel [14]:



where LI is the labelling index of the whole population and LI_P _is the labelling index of proliferating cells. If the number of labelled cells traversing the cell cycle per unit time is constant, we have



where T_C _is the duration of the cell cycle (T_C _=  + T_S _+ ). The LI can be calculated by the formula proposed by Johansson et al. [13]:



where n_t _is the total number of BrdUrd-positive and BrdUrd-negative cells measured per sample, G_2 _is the total number of G_2 _phase cells and G_2_^ul ^is the number of unlabelled G_2 _cells. In the proposed method, t < T_S _and G_2_^ul ^is negligible. Thus, the total number of G_2 _phase cells is approximately equal to G_2_^lu^.

Formulae for estimation of durations of cell cycle phases based on a decreasing exponential cell age distribution hypothesis are presented in the Additional file 1.

### RM approach

In addition to using the proposed formulae, T_S _values were estimated also using the classical RM method [1], here referred to as RM (linear), and the more recently proposed RM method, based on a cubic fit to the RM [6], here referred to as RM (cubic). The latter method also allows calculation of . Using the RM (linear) method, the relative mean DNA content RM(t) of the moving cohort of BrdUrd-labelled cells in relation to that of G_1 _and G_2 _cells, was calculated using the formula



where  and  are the mean DNA content of cells in G_1 _and G_2 _phases, respectively, F_LU _is the mean DNA content of labelled undivided cells (S^lu ^and G_2_^lu^) and t is post-labelling time. T_S _was calculated from the equation



Using the RM (cubic) method, T_S _was calculated from the equation



In this equation, ν is defined as



where f^lu^(t) and f^ld^(t) are fractions of labelled undivided and labelled divided cells, respectively, at post-labelling time t. In our case, f^lu^(t) is the fraction of S^lu ^and G_2_^lu ^cells, and f^ld^(t) – the fraction of G_1_^ld ^and S^ld ^cells, respectively, in the total number of cells n_t _measured per sample.

An assumed exponential growth rate c was estimated as



where



 was calculated from the equation

 = t + ln(1 - f^ld^(t)/2)/c     (19)

### Statistics

Pearson's correlation coefficient was used to determine the correlation between the T_S _or  values obtained using the proposed method and the RM approach. Differences between the GF values at day 4 and day 10 were analysed by the Wilcoxon rank sums test.

## Results

**Figure 3:**
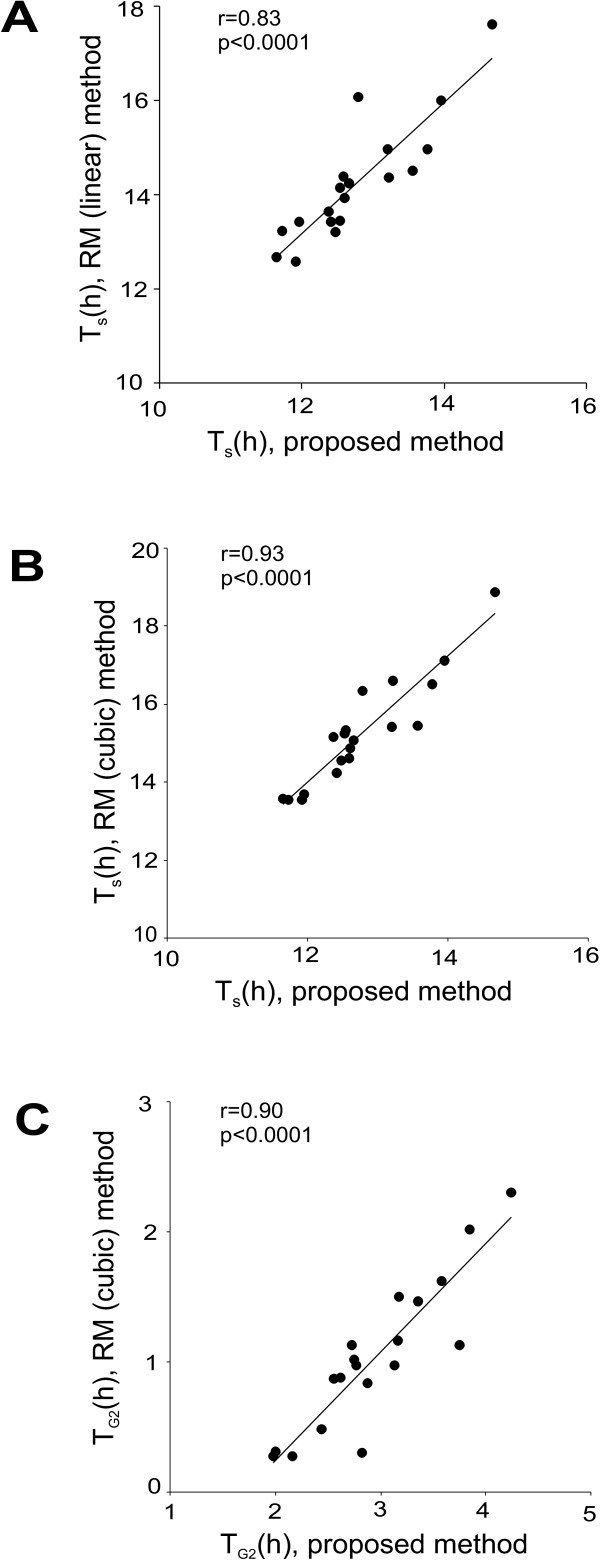
**Correlation between cell cycle phase duration values obtained with the proposed method and with the RM approach for mouse solid SL2 tumours**. A. Correlation between T_S _values obtained with the proposed method and with the RM (linear) method. B. Correlation between T_S _values obtained with the proposed method and with the RM (cubic) method. C. Correlation between  values obtained with the proposed method and with the RM (cubic) method.

To illustrate application of the proposed formulae, duration of cell cycle phases and GF were analysed in solid SL2 tumours at 4 and 10 days after implantation. , T_S_,  and T_C _values estimated using the proposed method and T_S _and  values in the same tumours estimated using the RM approach are given in Table 1. A significantly positive correlation between the T_S _values obtained using the proposed method and the RM approach was observed (Figures 3A and 3B). In addition, a significantly positive correlation was observed between the  values obtained with the proposed method and with the RM (cubic) method (Figure 3C).

**Figure 4:**
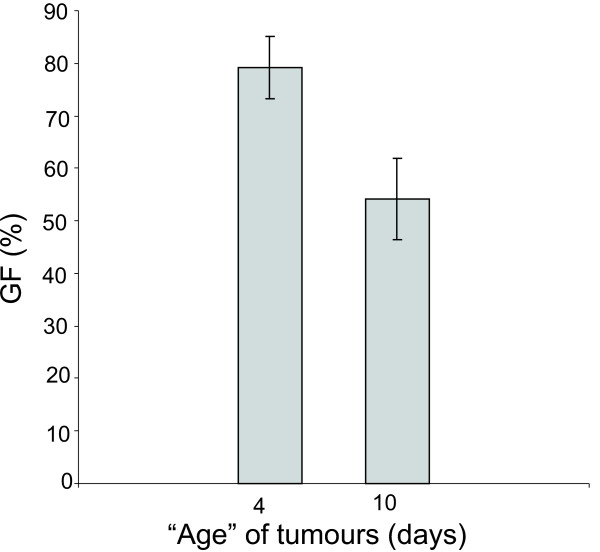
**GF in solid SL2 tumours at 4 days and at 10 days after tumour implantation (mean ± SD)**. The difference between GF at day 4 and day 10 is statistically significant (p = 0.0003).

GF in solid SL2 tumours at 4 days and 10 days after implantation, estimated using the proposed method, are shown in Figure 4. GF in solid SL2 tumours at 10 days was significantly lower than GF at 4 days after implantation.

**Figure 5:**
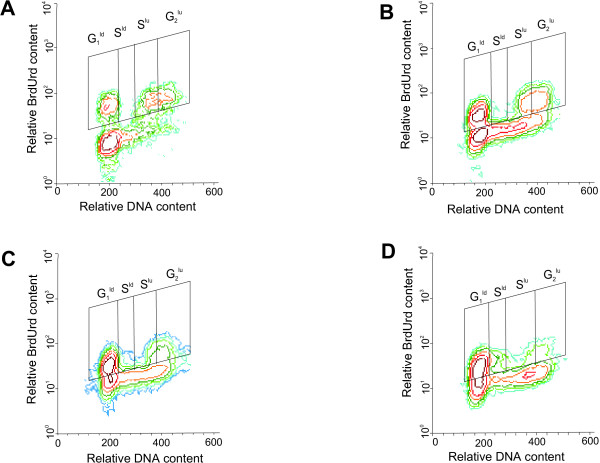
**Representative dot plots of BrdUrd content (log FITC fluorescence) vs. DNA content (propidium iodide fluorescence) of Jurkat cells at different post-labelling times**. Jurkat tumour cells were labelled with BrdUrd for 30 min and then fixed after 8 h (A), 10 h (B), 12 h (C) and 16 h (D). A. Labelled cells have not yet entered the S^ld ^compartment. B. Labelled cells start entering the S^ld ^compartment. C. Considerable proportions of labelled cells are present both in the S^ld ^and in the S^lu ^compartment. D. Labelled cells are leaving the S^lu ^compartment. The optimal time for measurement of cell kinetics in Jurkat cells with the proposed method appears to be 12 h.

Representative dot plots of BrdUrd content vs. DNA content of Jurkat cells at different post-labelling times are shown in Figure 5. Samples of Jurkat cells taken at 8 h after the BrdUrd pulse were not suitable for analysis with the proposed method, because there were no labelled cells in the S^ld ^compartment, i.e. the labelled cells had not yet entered the new S phase. Interval of 12 h appeared to be the most appropriate for cell kinetic analysis of Jurkat cells with the proposed method, because there were considerable proportions of cells both in the S^ld ^compartment and in the S^lu ^compartment. At 10 h, the number of labelled cells in the S^ld ^compartment was still rather low, whereas at 16 h, only the small number of cells remained in the S^lu ^compartment. Estimates of cell cycle phases and GF in Jurkat cell line obtained at 10 h, 12 h and 16 h after BrdUrd labelling are presented in Table 2.

Estimates of durations of cell cycle phases based on rectangular age distribution hypothesis and corresponding estimates obtained by calculations based on a decreasing exponential cell age distribution hypothesis are compared in supplementary Table 1 [see Additional file 1]. Numerical examples illustrating that the estimates of durations of cell cycle phases are not very sensitive to erroneous setting (+/-5%) of regions of interest in the DNA-BrdUrd plots are presented in supplementary Table 2 [see Additional file 2].

## Discussion

The proposed method is based on two assumptions. The first assumption, i.e., the labelled cell mortality or exit from the cell cycle during the period of measurement is negligible, can be regarded as reasonably simple and generally acceptable. On the other hand, it is quite difficult to obtain reliable estimates of cell death rates in bivariate BrdUrd/DNA cytometry. A method for estimating the rate of cell death in G2/M phase of exponentially growing cell populations has only recently been published [15].

The second assumption is that the number of labelled cells traversing the cell cycle per unit time is constant. Although rather simplistic, this assumption does not appear to induce a great extent of error, as illustrated by corresponding estimates obtained by calculations based on a decreasing exponential cell age distribution hypothesis.

Our results illustrate, that the proposed method can be successfully used to obtain kinetic estimates of the cell cycle and GF in a murine tumour in vivo and in human tumour cells in vitro. The finding of a decrease in GF with the "age" of murine tumours shows that information retrieved with the proposed method may be biologically relevant. A good correlation was found between the T_S _values of SL2 tumours obtained using our proposed method and the generally accepted RM (linear) or RM (cubic) methods. The  values of SL2 tumours obtained with the proposed method correlated with the  values obtained with the RM (cubic) method.

The T_S _values obtained with the proposed method had a narrower spread (11.6 to 14.7 h) compared to the T_S _values in the same tumours obtained with the RM (linear) and the RM (cubic) method (12.6 to 17.6 h and 13.5 to 18.9 h, respectively). Thus, the T_S _values of SL2 tumours obtained with the proposed method are more compatible with the notion that T_S _may be a species-specific constant [16]. The T_S _values obtained using the proposed method were slightly lower than those obtained using the RM methods. This difference can be explained by the fact that in the RM approach, the moving cohort of BrdUrd-labelled cells includes some G_2 _cells, which actually are not moving in the histogram. Thus, the RM of the cohort of BrdUrd-labelled cells is slower than actual traversing of cells through the S phase. Therefore, we think that our proposed formulae provide more accurate estimation of T_S_.

Similarly to other methods of analysis of cell kinetics using DNA profiles, the proposed method is subject to errors of profile interpretation. Using a fitting programme to extract more accurate data would be preferable. To our knowledge, however, currently available fitting programmes (including ModFit LT™, version 3.1) do not allow the separation of S^lu ^and S^ld ^compartments, the essential procedure in the proposed method. Thus, none of the currently available software can by applied for analysis of the DNA profile in a way required for the proposed method. On the other hand, the identification of a single channel with the minimum event count to separate S^lu ^and S^ld ^compartments was feasible in all DNA histograms analysed and, therefore, can be regarded as quite robust procedure.

Both the RM approach and the proposed method entail the problem of separation of diploid tumour cells from normal intratumoural cells. In the proposed method, admixture of normal cells does not affect the determination of T_C_, because this parameter is estimated using only BrdUrd-labelled cells, and proliferation of normal intratumoural cells is usually negligible. However, the proposed method may underestimate GF in diploid tumours, if procedures to separate normal cells from tumour cells are not used. The possible solutions to tackle this problem have been summarized by Antognoni P et al. [2] and are mainly based on using cytokeratin markers to separate epithelial tumour cells from intratumoural inflammatory cells and fibroblasts. In addition, it is recommended to present cell kinetic data from diploid and aneuploid tumours separately [17]. SL2 was proved to be an aneuploid (nearly tetraploid) tumour with the DNA index of 1.8 (data not shown). Thus, admixture of normal cells in our analysis can be ruled out.

In experiments with SL2 tumour, PI fluorescence was collected in the FL2 detector and compensation for the spectral overlap with FITC fluorescence was used. Generally, however, it is recommended to use FL3 (as done in experiments with Jurkat cells) rather than FL2 to overcome any crossover with the FITC in FL1 [12]. Running the samples at a higher FL3 voltage such that more of the scale is used would facilitate detection of hypodiploid DNA peaks.

In the RM approach, BrdUrd is injected usually 4 to 8 h prior to biopsy or surgical removal of a tumour [18]. The proposed method requires that considerable proportions of cells be found in each of the four compartments to be analysed (S^lu^, G_2_^lu^, G_1_^ld^, and S^ld^). This condition is fulfilled only if the time interval from the BrdUrd pulse to the time of measurement is longer than duration of G_2 _and G_1 _phases but still shorter than duration of S phase. Obviously, this cannot be assured in advance. Thus, the proposed method has to be tailored for a particular tumour type and the optimal time of measurement has to be found by trial and error. As an example of this trial and error procedure, the finding of an optimal time for measurement of cell kinetics in Jurkat cells is described in this paper.

Measurements with Jurkat cells also show, that the proposed method may be sensitive to intercell variability in phase transit times. If the time of measurement is too short and only the fastest cells enter S^ld ^compartment (e.g. 10 h in case of Jurkat cells), the duration of G_1 _phase may be underestimated. On the other hand, if the time of measurement is too long and only the slowest cells remain in the S^lu ^compartment (e.g. 16 h in case of Jurkat cells), the duration of S and other cell cycle phases may be overestimated. Thus, the time for measurement of cell kinetics with the proposed method has to be selected carefully. To minimize the effect of intercell variability in phase transit times, it is advisable to use the same time interval from the BrdUrd pulse to the time of measurement in all samples to be compared.

Measurements with cultured Jurkat cells presented in this paper suggest that the proposed method might be also suitable for studies on human tumours. It seems that the suitability of the proposed method for clinical studies could only be compromised if the G_1 _phase in human tumours in vivo was very long. Obviously, this can only be determined by testing the proposed method in clinical studies after infusion of BrdUrd to patients.

## Conclusion

The proposed method is relatively simple and permits estimation of cell cycle parameters, including T_C _and GF, from a single tumour sample after labelling with BrdUrd. We have shown that this method may be useful in preclinical studies, allowing estimation of changes in GF during growth of murine tumours. Experiments with human Jurkat cells suggest that the proposed method might also prove suitable for measurement of cell kinetics in human tumours. Development of suitable software enabling more objective interpretation of the DNA profile in this method would be desirable.

## Competing interests

The author(s) declare that they have no competing interests.

## Authors' contributions

RE conceived the mathematical formulae and contributed to elaboration of the manuscript. DC coordinated the study and wrote the manuscript. RJ analysed the data and participated in writing of the manuscript. NK performed flow cytometric analysis. VP contributed to elaboration of the manuscript. MM coordinated animal experiments, ethical guidelines. WDO critically appraised the manuscript. All authors read and approved the final manuscript.

## Pre-publication history

The pre-publication history for this paper can be accessed here:



## Supplementary Material

Additional file 1 Calculations of durations of cell cycle phases based on hypothesis of decreasing exponential cell age distribution. The Additional file 1 includes supplementary Table 1 – Durations of cell cycle phases in murine solid SL2 tumours calculated using rectangular or decreasing exponential cell age distribution Click here for file

Additional file 2 Supplementary Table 2 – Consequences of erroneous measure of percentages of cells in regions of interest (ROI) in the DNA-BrdUrd plot on the estimates of cell cycle phase durations and GF Click here for file

## Figures and Tables

**Table 1:** Estimation of durations of cell cycle phases in murine SL2 tumours using the proposed method and the RM methods *

Tumour "age"	Method	T_G1_	T_S_	T_G2_	T_C_
4 days (n = 10)	Proposed	5.1 ± 0.4	12.6 ± 1.0	2.6 ± 0.5	20.3 ± 1.1
	RM (linear)	N.A.	14.1 ± 1.5	N.A.	N.A.
	RM (cubic)	N.A.	14.8 ± 1.6	0.7 ± 0.4	N.A.
10 days (n = 9)	Proposed	5.4 ± 0.5	12.9 ± 0.6	3.3 ± 0.5	21.6 ± 0.9
	RM (linear)	N.A.	14.4 ± 1.1	N.A.	N.A.
	RM (cubic)	N.A.	15.8 ± 3.3	1.4 ± 0.6	N.A.

* Values are given in h (mean ± SD)

N.A. = not available

**Table 2:** Estimation of durations of cell cycle phases and GF in Jurkat cell line using the proposed method at different times of measurement *

Hours after BrdUrd labelling	T_G1_	T_S_	T_G2_	T_C_	GF (%)
10	5.2	11.1	4.7	21.0	78.9
12	9.3	12.2	2.4	23.9	79.3
16	14.2	16.3	1.0	31.5	53.3

* Values obtained from individual measurements, given in h
