# Correction: a method to estimate cell cycle time and growth fraction using bromodeoxyuridine-flow cytometry data from a single sample

**DOI:** 10.1186/1471-2407-6-184

**Published:** 2006-07-11

**Authors:** Rimantas Eidukevicius, Dainius Characiejus, Ramunas Janavicius, Nijole Kazlauskaite, Vita Pasukoniene, Mykolas Mauricas, Willem Den Otter

**Affiliations:** 1 Faculty of Mathematics and Informatics, Vilnius University, Naugarduko 24, 03225 Vilnius, Lithuania; 2 Institute of Oncology, Vilnius University, Santariškių 1, 08660 Vilnius, Lithuania; 3 Institute of Immunology, Vilnius University, Moletų pl. 29, 08409 Vilnius, Lithuania; 4 Department of Pathobiology, Utrecht University, P.O.Box 80158, 3508 TD Utrecht, The Netherlands

In our article in BMC Cancer [1] we modelled our graph for the passage of bromodeoxyuridine-labelled cells through the cell cycle presented in Figure 1 on the figure published by Johansson et al. [2]. We regret that we failed to quote this article and we are grateful to its authors and the editors for bringing this matter to our attention.

## Pre-publication history

The pre-publication history for this paper can be accessed here:


