# Supplementary material for: A method to estimate cell cycle time and growth fraction using bromodeoxyuridine-flow cytometry data from a single sample
Source: BMC Cancer. 2005 Sep 22;5:122. doi: 10.1186/1471-2407-5-122 (PMC1261259; doi:10.1186/1471-2407-5-122)
Supplement: Additional file 1 — Calculations of durations of cell cycle phases based on hypothesis of decreasing exponential cell age distribution. The Additional file 1 includes supplementary Table 1 – Durations of cell cycle phases in murine solid SL2 tumours calculated using rectangular or decreasing exponential cell age distribution [file 1471-2407-5-122-S1.doc]

## Calculations of durations of cell cycle phases based on hypothesis of decreasing exponential cell age distribution

Calculations presented below are based on the decreasing exponential cell age distribution hypothesis proposed by Steel [14]. This model assumes exponential growth of cell population with no cell loss, in which proliferating cells have a uniform cell cycle time T_C_. The parameter a is used for the average number of proliferating daughter cells produced at each division. In this model $\text{a}\in\text{(1, 2]}$.

The density function for cells of age *τ* is

$$f(\tau)=\frac{a}{a-1}b\exp(-b\tau),\text{ }0\leq\tau\leq T_{C},\text{ }b=\frac{\ln(a)}{T_{C}}.$$

The proportion of cells of age interval [s_1_, s_2_] is

$$F(s_{2})-F(s_{1})=\int_{s_{1}}^{s_{2}} f(s)ds=\frac{a}{a-1}(\exp(-bs_{1})-\exp(-bs_{2})),\text{ }s_{1}\leq s_{2}.$$

According to the non-rectangular cell age distribution hypothesis, the following equations may be written:

$$\begin{matrix} S^{lu}=n_{0}(F(T_{G1}+T_{S}-t)-F(T_{G1})), \\ G_{2}^{lu}+\frac{1}{2}(G_{1}^{ld}+S^{ld})=n_{0}(F(T_{G1}+T_{S})-F(T_{G1}+T_{S}-t)), \\ \frac{1}{2}(G_{1}^{ld}+S^{ld})=n_{0}(F(T_{G1}+T_{S})-F(T_{G1}+T_{S}-t+T_{G2})), \\ \frac{1}{2}S^{ld}=n_{0}(F(T_{G1}+T_{S})-F(T_{G1}+T_{S}-t+T_{G2}+T_{G1})) \end{matrix}$$

where n_0_ is the number of cells at the moment of labelling ($\text{t}=0$). According to the formula proposed by Johansson et al. [13]

$$n_{0}=n_{t}-\frac{1}{2}(S^{ld}+G_{1}^{ld})-G_{2}^{lu}$$

Solving this system of equations, the durations of cell cycle phases and values of the parameter a can be calculated. Cell kinetic estimates of 5 SL2 tumours obtained by calculations based on rectangular or decreasing exponential cell age distribution hypothesis are given in supplementary Table 1.

## Supplementary Table 1 - Durations of cell cycle phases in murine solid SL2 tumours calculated using rectangular or decreasing exponential cell age distribution

| Tumour No. | Rectangular distribution | | | Decreasing exponential distribution | | | |
| --- | --- | --- | --- | --- | --- | --- | --- |
|  | T_G1_ | T_S_ | T_G2_ | T_G1_ | T_S_ | T_G2_ | a * |
| 1 | 5.0 | 11.9 | 2.7 | 4.3  4.8 | 16.7  11.1 | 4.4  1.9 | 0.0  6.2 |
| 2 | 4.9 | 13.6 | 2.8 | 4.3  4.8 | 33.4  12.5 | 4.2  2.2 | 0.0  3.2 |
| 3 | 4.4 | 14.7 | 3.8 | 4.5 | 12.2 | 2.4 | 13.7 |
| 4 | 5.6 | 12.0 | 2.0 | 5.3 | 11.2 | 1.4 | 4.8 |
| 5 | 5.7 | 11.7 | 2.0 | 5.0 | 10.8 | 1.1 | 12.8 |

Values of T_G1_, T_S_, T_G2_ and T_C_ are given in h

* Biologically meaningless values of the parameter a may be due to violation of the model assumptions. The solution of the system may be reduced to the solution of the quadratic equation. Two lines in a cell correspond to two positive solutions
