# Supplementary material for: A method to estimate cell cycle time and growth fraction using bromodeoxyuridine-flow cytometry data from a single sample
Source: BMC Cancer. 2005 Sep 22;5:122. doi: 10.1186/1471-2407-5-122 (PMC1261259; doi:10.1186/1471-2407-5-122)
Supplement: Additional file 2 — Supplementary Table 2 – Consequences of erroneous measure of percentages of cells in regions of interest (ROI) in the DNA-BrdUrd plot on the estimates of cell cycle phase durations and GF [file 1471-2407-5-122-S2.doc]

## Supplementary Table 2 - Consequences of erroneous measure of percentages of cells in regions of interest (ROI) in the DNA-BrdUrd plot on the estimates of cell cycle phase durations and GF*

| Tumour  No. | Measurements | | T_G1_ | T_S_ | T_G2_ | T_C_ | GF (%) |
| --- | --- | --- | --- | --- | --- | --- | --- |
| 1 | Primary data | | 5.0 | 11.9 | 2.7 | 19.6 | 81.9 |
|  | 5% error | G_1_🡪S^lu^ S^ld^🡪G_2_ | 5.2 | 12.0 | 2.6 | 19.8 | 82.7 |
|  |  | G_1_🡨S^lu^  S^ld^ 🡨G_2_ | 4.7 | 11.8 | 2.9 | 19.4 | 81.0 |
|  |  | G_1_ 🡪 S^lu^  S^ld^ 🡨G_2_ | 5.1 | 11.8 | 2.8 | 19.7 | 85.2 |
|  |  | G_1_🡨S^lu^  S^ld^ 🡪G_2_ | 4.9 | 12.1 | 2.7 | 19.6 | 78.7 |
| 2 | Primary data | | 5.6 | 12.0 | 2.0 | 19.6 | 81.2 |
|  | 5% error | G_1_🡪S^lu^ S^ld^🡪G_2_ | 5.9 | 12.0 | 1.9 | 19.8 | 82.9 |
|  |  | G_1_🡨S^lu^  S^ld^ 🡨G_2_ | 5.4 | 11.9 | 2.1 | 19.4 | 79.6 |
|  |  | G_1_ 🡪 S^lu^  S^ld^ 🡨G_2_ | 5.8 | 11.8 | 2.0 | 19.6 | 84.4 |
|  |  | G_1_🡨S^lu^  S^ld^ 🡪G_2_ | 5.5 | 12.1 | 1.9 | 19.5 | 78.1 |

## Supplementary Table 2 (continued)

* Data of murine SL2 tumours

| Tumour  No. | Measurements | | T_G1_ | T_S_ | T_G2_ | T_C_ | GF (%) |
| --- | --- | --- | --- | --- | --- | --- | --- |
| 3 | Primary data | | 4.8 | 13.2 | 4.3 | 22.3 | 48.4 |
|  | 5% error | G_1_🡪S^lu^ S^ld^🡪G_2_ | 5.1 | 13.4 | 4.0 | 22.5 | 48.7 |
|  |  | G_1_🡨S^lu^  S^ld^ 🡨G_2_ | 4.6 | 13.1 | 4.5 | 22.1 | 48.0 |
|  |  | G_1_ 🡪 S^lu^  S^ld^ 🡨G_2_ | 4.9 | 12.9 | 4.3 | 22.1 | 50.3 |
|  |  | G_1_🡨S^lu^  S^ld^ 🡪G_2_ | 4.8 | 13.5 | 4.2 | 22.5 | 46.4 |
| 4 | Primary data | | 4.7 | 12.5 | 3.9 | 21.1 | 57.4 |
|  | 5% error | G_1_🡪S^lu^ S^ld^🡪G_2_ | 5.0 | 12.7 | 3.7 | 21.3 | 57.8 |
|  |  | G_1_🡨S^lu^  S^ld^ 🡨G_2_ | 4.5 | 12.4 | 4.0 | 21.0 | 57.1 |
|  |  | G_1_ 🡪 S^lu^  S^ld^ 🡨G_2_ | 4.8 | 12.3 | 3.9 | 21.0 | 60.0 |
|  |  | G_1_🡨S^lu^  S^ld^ 🡪G_2_ | 4.7 | 12.8 | 3.8 | 21.2 | 55.1 |

Values of T_G1_, T_S_, T_G2_ and T_C_ are given in h

G_1_, S^lu^, S^ld^, G_2_ = corresponding ROI in the DNA-BrdUrd plot

🡪, 🡨 arrows indicate corresponding shift of 5% of cells from one ROI to another
